# Overlap of Autism and Seizures: Understanding Cognitive Comorbidity

**DOI:** 10.4103/0973-1229.58823

**Published:** 2010

**Authors:** Neha Khetrapal

**Affiliations:** * *[M.A. Cognitive Science] Centre of Excellence “Cognitive Interaction Technology” (CITEC) And Faculty of Psychology & Sport Sciences, University of Bielefeld, Bielefeld, Germany 33615*

**Keywords:** *Hippocampus*, *Amygdala*, *DSM*, *Brain*, *Intervention*, *Epilepsy*, *Autism*, *Comorbidity*, *Cognitive comorbidity*

## Abstract

This article introduces the concept of ‘cognitive comorbidity,’ which lays emphasis on common cognitive deficits that cut across different disorders. The concept is illustrated with the help of two commonly reported overlapping conditions (autism and epilepsy). It is further explained by concentrating on two important cognitive processes of facial emotional recognition and emotional memory, shown to be compromised in both conditions; and their underlying neural substrates. Cognitive comorbidity is then contrasted with ‘comorbidity,’ a term which is more commonly used for describing cognitive disorders. The paper closes by providing directions for rehabilitative and theoretical efforts that could be inspired by the newly introduced concept.

## Introduction

This article aims to introduce and understand the concept of ‘cognitive comorbidity’ and differentiate it from the more common usage of the term ‘comorbidity’. The current paper strives to achieve this aim by describing the overlapping symptoms of autism and epilepsy. These two disorders are chosen as examples because of the high rate of association between the two (Giovanardi-Rossi, Posar & Parmeggiani, 2000). This article is not an attempt to discuss the comorbidity between these two disorders in the technical sense of the word “comorbidity,” as it is described in scientific literature but to discuss and highlight the overlap of cognitive deficits. All through, the focus will be on cognitive processes and references to brain/neural structures are provided to advance understanding of the concept. This way of explanation is hoped to be a better method from the viewpoint of theoretical formulations and would also help to throw light on the issue of “comorbidity,” the way it is described in the scientific literature. Therefore, first the description of comorbidity is given, which is followed by a brief introduction to the disorders.

## Comorbidity, Autism & Epilepsy

Fried, Ferrucci, Darer, Williamson & Anderson (2004) explain comorbidity as, “the concurrent presence of two or more medically diagnosed diseases in the same individual, with the diagnosis of each contributing disease based on established, widely recognized criteria”.

The DSM-IV defines autism as a disorder with impairments in socialization, imagination and communication with stereotyped repetitive interests (American Psychological Association, 1995). Autism is a prototypical example of autism spectrum disorders (ASD). The term ASD is used to define life-long developmental disorders of the brain with variable severity (Tuchman & Rapin, 2002). Approximately one-third of the children on the autism spectrum are shown to develop epilepsy (Volkmar & Cohen, 1991; see also, Tuchman, 2001).

Epilepsy is defined as two unprovoked seizures of any kind that result from genetic defects, due to diffuse or focal pathology in the brain, or due to an unknown cause. Thus seizures due to trauma, infection or metabolic illness are not defined as epilepsy (Tuchman & Rapin, 2002).

## Neural Speculations

The neurobiology of autism and epilepsy, especially temporal lobe epilepsy, can be similar; that is, the integrity of the amygdala has been found to be compromised in both the disorders; and various cognitive tasks conducted on people inflicted by both of these separately reflect this compromised integrity (Brierley *et al*. 2004; Beversdorf *et al*. 1998; Paesschen *et al*. 1996). Two most important tasks that have been conducted to demonstrate the cognitive deficits are facial emotion recognition and emotional memory task. Facial emotion recognition and emotional memory are hypothesized to be dependent upon the integrity of functional connections between amygdala and the fusiform cortex (Vuilleumier, Richardson, Armony, Driver & Dolan, 2004) and between amygdala and the hippocampus respectively (Phelps, 2004). Consistent with the hypothesized relations, Brierley *et al*. (2004) showed inefficient performance by patients suffering from epilepsy and amygdala damage on the two tasks. Their emotion recognition task dealt with perception of affect from faces and voices while the emotional memory tasks dealt with narratives and novel word recognition. There was also a lack of association between the emotional memory and the emotion recognition tasks, thus showing that these two are different cognitive processes.

Brierley *et al*. (2004) also proposed two different neural connections responsible for supporting performance on the two tasks where deficits in facial emotion recognition could arise due to disruptions of connections from the amygdala to the fusiform cortex, and problems on the emotional memory task could arise due to disruptions of connections from the amygdala and hippocampus. Similar performance deficits are also shown by the autistic subjects of Beversdorf *et al*. (1998) on an emotional memory task. Beversdorf and colleagues presented emotional sentences and coherent/incoherent stories and word lists to their autistic and normal subjects. Autistic and normal individuals showed comparable performance on recalling coherent/incoherent word lists and stories but differed only with respect to recalling emotional sentences, with the autistics remembering lesser number of emotional sentences. Deficits of emotional facial recognition have also been observed for autistics where they showed normal performance on a facial discrimination task but impaired performance on emotional facial recognition task (Ashwin *et al*. 2006). These results put together show that the two cognitive processes can be disturbed in both the disorders.

## Implications of Cognitive Comorbidity

This paper makes an initial attempt to highlight the common cognitive processes that might be disturbed in both the conditions giving a possible clue to the comorbidity, in this case, cognitive comorbidity. The paper does not go into the details of different types of autistic spectrum problems or different types of epilepsies as the aim is to highlight broad cognitive processes that could be disturbed. It only concentrates on the common neural and cognitive substrates. By highlighting the issue of cognitive comorbidity, the paper is a step in the direction of encouraging researchers to investigate such issues by looking at the overlap of cognitive deficits between disorders and not to emphasize “comorbidity” based on the incidence rate of the disorders.

**Figure 1:**
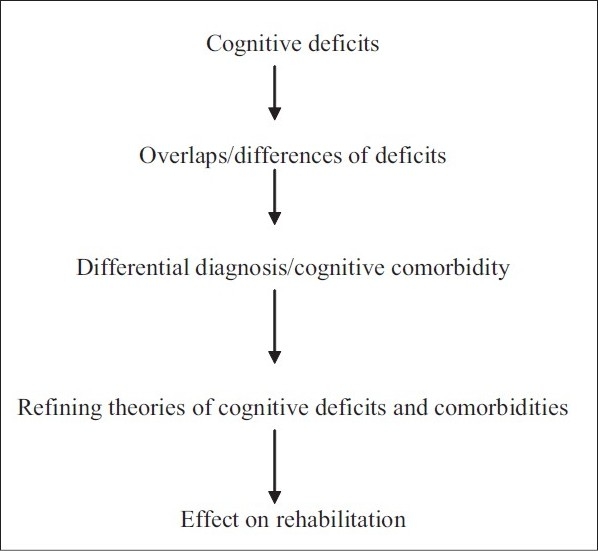
Flow chart of the paper

## Implications for Future Research

The previous sections show that emotional memory and emotional facial recognition could be disturbed in a similar manner in both the disorders despite the fact that these two disorders are totally different from each other. In order to provide direct support for the concept of cognitive comorbidity, we need studies that concentrate on examining the performance of both autistics and individuals with epilepsy on these tasks in the same study so that direct comparisons could be drawn. In its current state, the concept of cognitive comorbidity could only be indirectly supported with findings from separate studies and thus future studies need to be planned not only on these two disorders but other similar cases of disorders that have been reported in the literature as comorbid or associated disorders; for instance, attention deficit hyperactivity disorder (ADHD) and tics.

Research directed at theories of cognitive disorders and cognitive rehabilitation would save precious efforts if guided by the concept of cognitive comorbidity as this would discourage separate research attempts focusing on different disorders and would definitely go a long way in encouraging coordinated efforts at understanding the overlapping cognitive deficits that cut across the different categories of disorders. It becomes a lot easier to explain the nature of cognitive functions that are disrupted in a similar manner across different disorders but it is difficult to explain these disruptions across two different disorders especially when the current thrust of the field is to place disorders into separate categories. Emphasizing on cognitive comorbidity also helps one apply precious successful attempts of rehabilitation designed to address an underlying cognitive deficit across the disorders but this can only be done when it is known that similar cognitive processes are disturbed. To come up with different rehabilitation programs for different disorders can be very costly, especially if one follows the strategy of placing these into different categories as this can happen at the cost of ignoring the common underlying cognitive substrates.

## Conclusions

Current article highlights the concept of cognitive comorbidity by elucidating the differences and similarities between autism and epilepsy. The hypothesis still requires direct comparison of patients suffering from both autism and epilepsy on the commonly disturbed processes that support emotional memory and emotional facial recognition. Direct comparison in this manner will not only be helpful in providing support for the cognitive comorbidity hypothesis but will also help us understand the common underlying symptoms that might be important and hence could be targeted commonly across the disorders. This will help us save previous time and resources deployed for treating different disorders separately. For instance, if research shows that emotional memory and facial emotional discrimination are commonly disturbed in both autism and epilepsy, one single intervention strategy (e.g. cognitive rehabilitation program) could be easily designed for autistics and patients of epilepsy. Providing support for cognitive comorbidity might not work well with researchers and clinicians who are looking for more discrimination among the disorders for the purpose of classification and distinction among various categories but this concept could potentially work well for therapists who deal with many patients and suffer from time constraints. Efforts directed at cognitive comorbidity will work well with patients and their families who are not happy to be tagged by some diagnostic label or the other.

It is hoped that future work in this direction can be instrumental in stimulating research and highlighting other such overlaps that will be ultimately useful in constraining theories about cognitive processes and also trimming diagnostic criteria and saving precious resources geared towards interventions.

### Take Home Message

This article makes an attempt to highlight the cognitive comorbidity between autism and epilepsy where the structural and the functional integrity of the amygdala is compromised. Hence, there are similar cognitive deficits in both the disorders observed on two tasks, namely, facial emotion recognition and emotional memory, which are supported by the amygdala. Work also shows that these two cognitive processes are not correlated with each other and either both or one of them can be disturbed in autism

### Conflict of Interest

None declared.

### Declaration

This work or parts of it are not published anywhere else.

## Questions that the Paper Raises

1. How can the concept of cognitive comorbidity be extended to other cognitive disorders?
2. How can clinicians save on precious resources by developing the concept of common disturbance among disorders and develop intervention techniques accordingly?
3. How well can this concept of cognitive comorbidity be integrated into popular classifications like the DSM?
4. Should we altogether abolish placing cognitive disorders into various classifications and only focus on overlapping cognitive profiles?

## About the Author


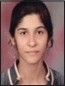
 *Neha Khetrapal is currently a graduate student at the Centre of Excellence “Cognitive Interaction Technology” (CITEC) and the Faculty of Psychology and Sport Sciences, investigating the interaction of spatial processes and language and is supported by Deutsche Forschungsgemeinschaft (DFG) grant managed through the Graduate School of CITEC, Germany. She has been a holder of various awards, and the most important recognition earned by her is from Marquis Who’s Who in the World for 2009. The author also serves currently as the reviewer for Journal of Social and Psychological Sciences http://www.jspsciences.org and as the editorial member of the New School of Psychology Bulletin www.nspb.net*
